# Attitudes and behaviour predict women's intention to drink alcohol during pregnancy: the challenge for health professionals

**DOI:** 10.1186/1471-2458-11-584

**Published:** 2011-07-22

**Authors:** Elizabeth Peadon, Janet Payne, Nadine Henley, Heather D'Antoine, Anne Bartu, Colleen O'Leary, Carol Bower, Elizabeth J Elliott

**Affiliations:** 1 Discipline of Paediatrics and Child Health, Sydney Medical School, The University of Sydney, The Children's Hospital at Westmead, Locked Bag 4001, Westmead, NSW 2145, Australia; 2 Telethon Institute for Child Health Research, Centre for Child Health Research, The University of Western Australia, PO Box 855, West Perth, Western Australia 6872, Australia; 3 Centre for Applied Social Marketing and Research, Edith Cowan University, 270 Joondalup Drive, Joondalup, Western Australia 6027, Australia; 4 School of Nursing and Midwifery, Faculty Health Sciences, Curtin University of Technology, GPO Box U1987, Perth, Western Australia 6845, Australia

## Abstract

**Background:**

To explore women's alcohol consumption in pregnancy, and potential predictors of alcohol consumption in pregnancy including: demographic characteristics; and women's knowledge and attitudes regarding alcohol consumption in pregnancy and its effects on the fetus.

**Methods:**

We conducted a national cross-sectional survey via computer assisted telephone interview of 1103 Australian women aged 18 to 45 years. Participants were randomly selected from the Electronic White Pages. Pregnant women were not eligible to participate. Quotas were set for age groups and a minimum of 100 participants per state to ensure a national sample reflecting the population. The questionnaire was based on a Health Canada survey with additional questions constructed by the investigators. Descriptive statistics were calculated and logistic regression analyses were used to assess associations of alcohol consumption in pregnancy with participants' characteristics, knowledge and attitudes.

**Results:**

The majority of women (89.4%) had consumed alcohol in the last 12 months. During their last pregnancy (n = 700), 34.1% drank alcohol. When asked what they would do if planning a pregnancy (n = 1103), 31.6% said they would consume alcohol and 4.8% would smoke. Intention to consume alcohol in a future pregnancy was associated with: alcohol use in the last pregnancy (adjusted OR (aOR) 43.9; 95% Confidence Interval (CI) 27.0 to 71.4); neutral or positive attitudes towards alcohol use in pregnancy (aOR 5.1; 95% CI 3.6 to 7.1); intention to smoke in a future pregnancy (aOR 4.7; 95% CI 2.5 to 9.0); and more frequent and higher current alcohol consumption.

**Conclusions:**

Women's past pregnancy and current drinking behaviour, and attitudes to alcohol use in pregnancy were the strongest predictors of alcohol consumption in pregnancy. Targeted interventions for women at higher risk of alcohol consumption in pregnancy are needed to change women's risk perception and behaviour.

## Background

Alcohol is teratogenic and alcohol consumption during pregnancy may lead to growth restriction, birth defects, and structural and/or functional problems of the central nervous system in the exposed fetus [1,2]. Alcohol consumption during pregnancy is also associated with poor pregnancy outcomes such as miscarriage and premature labour [3]. Fetal Alcohol Spectrum Disorders (FASD) is the umbrella term for the range of diagnoses which may result in a fetus who has been exposed to alcohol during pregnancy. These diagnoses include Fetal Alcohol Syndrome, Alcohol Related Neurodevelopmental Disorder and Alcohol Related Birth Defects. FASD are potentially preventable causes of lifelong physical and mental disabilities [4].

Although reported rates of alcohol use among young women vary, the majority of young women in many countries consume alcohol. For example, 57.1% of women aged 18 to 25 years in the USA report current drinking [5], as do 86.7% of 20 to 29 year old Australian women [6], 87.3% of 20 to 24 year old Canadian women [7] and 79% of 16 to 24 year old UK women [8]. Variations in reported rates may be due to cultural factors and differences in methodology. Young women frequently report binge, risky and hazardous alcohol consumption [9-11]. Alcohol use can influence young women's sexual behaviour and is associated with risk of unplanned pregnancy [12,13], and 46% of pregnancies in Australian women have been reported to be unplanned [14]. Moreover, many women do not recognise pregnancy until the fourth to sixth week of gestation and in one study of pregnant women, up to 60% of women who drank alcohol in the periconception period did not know they were pregnant at four weeks' gestation [15]. Consequently, unintended fetal alcohol exposure is common.

In an Australian study, more than half the women reported drinking alcohol in at least one trimester of pregnancy [14]. In a large US study, one third of neonates had been exposed to alcohol during the pregnancy [16]. Some risk factors for alcohol consumption in the periconception period or pregnancy have been identified including increased maternal age[15], higher maternal education level [15-17], and maternal smoking [16-18]. In most studies of predictors of alcohol consumption in pregnancy, demographic characteristics have been examined. However, knowledge and attitudes are important influences of behaviour [19,20].

In order to design effective interventions to prevent fetal alcohol exposure and hence Fetal Alcohol Spectrum Disorders, we need to understand the potential influences on women's alcohol consumption during pregnancy and identify women who may be at higher risk and require targeted interventions. To address this gap, we analysed data from a cross-sectional national survey of women of child bearing age with regards to their previous alcohol consumption in pregnancy and intended consumption in a future pregnancy. We explored the associations between women's alcohol consumption and their demographic characteristics, and their knowledge and attitudes regarding alcohol use in pregnancy and its effects on the fetus.

## Methods

### Participants

A sample size of 1100 Australian women aged 18 to 45 years was set, with quotas to reflect the age distribution of Australian women in the 18 to 45 year age group in 2003 [21] and a minimum of 100 respondents from each state. A computer-generated random national sample was selected from the Electronic White Pages. To comply with the Ethics Committee's recommendation that the topic might generate anxiety, pregnant women were ineligible to participate.

### Questionnaire (Additional file 1: Alcohol and Pregnancy Questionnaire)

Our "Alcohol and Pregnancy Questionnaire" was based on the Health Canada survey "Alcohol Use During Pregnancy and Awareness of Fetal Alcohol Syndrome" [22] but modified for the Australian context, with additional questions on current alcohol consumption and previous alcohol consumption in pregnancy. A pilot study of 20 randomly selected women, conducted in August 2006, led to minor changes in the questionnaire. The questionnaire was administered as a Computer Assisted Telephone Interview in August and September 2006. We collected demographic data (age, ethnicity, parity, postcode, educational level, employment, marital status) and data on women's knowledge and attitudes regarding alcohol consumption in pregnancy and its effects on the fetus; alcohol consumption and smoking in the last pregnancy and intended practice in future pregnancy; and potential influences on alcohol consumption in pregnancy, including the influence of women's partners. Women were asked also asked about their current alcohol consumption, which was recorded in Australian standard drinks which contain 10 g of alcohol. The data on women's knowledge and attitudes have been reported previously [23]. Knowledge was ascertained through open questions and agreement with statements. Attitude was ascertained through agreement with statements and an open response to a scenario of seeing a pregnant woman drinking alcohol.

### Statistical analysis

Data were analysed using SPSS (version 15.0, SPSS Chicago, IL, USA). Descriptive statistics were calculated. Odds ratios (OR) and 95% Confidence Intervals (CI) were calculated for associations between demographic characteristics and alcohol consumption in previous pregnancy and intended alcohol consumption when planning a pregnancy and in a future pregnancy. Age, birth history and level of education were identified as potential confounders and adjusted odds ratios (aOR) were calculated using forward stepwise logistic regression. A sample size of n = 1100 provided estimates within 2% for variables with a prevalence of 40 to 50%.

### Ethics

The study protocol was approved by Ethics Committees of the Women's and Children's Health Service, Perth, Western Australia and The University of Sydney and the Western Australian Aboriginal Health Information and Ethics Committee.

## Results

### Respondent characteristics

1603 eligible women were contacted to acquire the planned sample size of 1100. 1103 eligible women (68.8%) provided consent and completed the interview. The characteristics of the respondents have been reported previously and are summarised here [23]. The mean age of women in the survey was 32 years, and 700 (63.5%) had given birth previously. The median time since the last pregnancy was 5 years (range: less than one year to 23 years). The majority of women (63.2%) were married or had a de facto or life partner and were employed (63.6%). Most women (79.4%) were born in Australia and 21 (1.9%) identified as Aboriginal or Torres Strait Islander. The majority of respondents (61.4%) had a post-school qualification. The characteristics of respondents were similar to those of women in the Australian population overall, except that respondents were more likely to have been born in Australia, to have had a previous pregnancy, and to have achieved a higher level of education; and less likely to identify as Aboriginal or Torres Strait Islander.

### Alcohol consumption and smoking in pregnancy (Table 1)

**Table 1:** Alcohol consumption and smoking (n = 1103)

Characteristic	n (%)
**Alcohol consumption and intended consumption in pregnancy**	
Drank alcohol in the last pregnancy (n = 700)*	239 (34.1)
Intend to drink alcohol if planning to become pregnant	348 (31.6)
Intend to drink alcohol if pregnant	261 (23.7)
**Smoking and intended smoking in pregnancy**	
Smoked in last pregnancy (n = 700)*	113 (16.1)
Intend to smoke if planning to become pregnant	53 (4.8)
Intend to smoke if pregnant	44 (4.0)
**Have ever tried alcohol**	1082 (98.1)
**Have had an alcoholic drink of any kind in the last 12 months**	986 (89.4)
**"In the last 12 months, how often did you have an alcoholic drink of any kind?"**	
Every day	39 (3.5)
5 to 6 days a week	32 (2.9)
3 to 4 days a week	149 (13.5)
1 to 2 days a week	313 (28.4)
2 to 3 days a month	159 (14.4)
about 1 day a month	119 (10.8)
Less often	175 (15.9)
Do not drink	117 (10.6)
**"On a day that you have an alcoholic drink, how many standard drinks do you usually have?"**	
13 or more drinks	7 (0.6)
11 to 12 drinks	4 (0.4)
7 to 10 drinks	47 (4.3)
5 to 6 drinks	103 (9.3)
3 to 4 drinks	265 (24.0)
1 to 2 drinks	526 (47.7)
less than 1 drink	34 (3.1)
Do not drink	117 (10.6)
**Maximum number of alcoholic standard drinks consumed in a day in the last 12 months**	
20 or more drinks	53 (4.8)
11 to 19 drinks	119 (10.8)
7 to 10 drinks	238 (21.6)
5 to 6 drinks	185 (16.8)
3 to 4 drinks	197 (17.9)
1 to 2 drinks	181 (16.4)
Less than 1 drink	13 (1.2)
Do not drink	117 (10.6)
**"How many alcoholic standard drinks did you have yesterday?"**	
0 drinks	829 (75.2)
1 to 2 drinks	181 (16.4)
3 to 4 drinks	54 (4.9)
5 or more drinks	39 (3.5)
**"Do you consider yourself:"**	
a non-drinker	127 (11.5)
an ex-drinker	17 (1.5)
an occasional drinker	301 (27.3)
a light drinker	227 (20.6)
a social drinker	400 (36.3)
a heavy drinker	13 (1.2)
a binge drinker	18 (1.6)
**"Do you consider yourself:"**	
Non-smoker	697 (63.2)
Ex-smoker	207 (18.8)
Smoker	199 (18.0)

* Women who had previously given birth (n = 700)

Among the women who had previously given birth (n = 700), 34.1% drank alcohol and 16.1% smoked during their last pregnancy. When women were asked what they would do if they were planning to become pregnant (n = 1103), just under a third said they would drink alcohol (31.6%) and 4.8% would smoke. When asked what they would do if they were pregnant (n = 1103), 23.7% of women would drink alcohol and 4.0% would smoke.

### Current alcohol and tobacco consumption (Table 1)

The majority of women had consumed alcohol in the past 12 months (89.4%), but a minority of women smoked (18%). Almost half the women drank alcohol every week (48.3%) and 19.9% drank alcohol on three or more days every week. Approximately half the respondents drank two standard drinks or less on a usual day when they consumed alcohol (50.8%), but 14.6% of women drank five or more standard alcoholic drinks on a usual day. Over half the women had consumed five or more standard alcoholic drinks on at least one day in the past twelve months (54%) and 15.6% had consumed 11 or more drinks on one day. When women were asked to identify their current drinking status from a list of terms, 13% identified as non-drinkers or ex-drinkers and 2.8% identified as heavy or binge drinkers.

### Partner's influence on alcohol consumption in pregnancy (Table 2)

**Table 2:** Partner influence on alcohol consumption in pregnancy (n = 1103)

	"If your spouse or partner continued to drink alcohol during your pregnancy?"	"If your spouse or partner encouraged you to stop or cut back your alcohol drinking during your pregnancy?"	"If your spouse or partner were to offer you alcohol during your pregnancy?"	"If your spouse or partner stopped drinking alcohol during your pregnancy?"
	n (%)	n (%)	n (%)	n(%)
Would be more likely to drink alcohol	44 (4.0)	30 (2.7)	67 (6.1)	7 (0.6)
Would be less likely to drink alcohol	61 (5.5)	422 (38.3)	109 (9.9)	336 (30.5)
Would make no difference to behaviour	998 (90.5)	651 (59.0)	927 (84.0)	760 (68.9)

Most women reported that their partners' behaviour would not influence their alcohol consumption in pregnancy. However, approximately one third said they would be less likely to drink alcohol in pregnancy if their partner encouraged them to stop or cut back (38.3%) or if their partner stopped drinking alcohol during the pregnancy (30.5%).

### Factors associated with alcohol use in pregnancy (Tables 3 and 4)

**Table 3:** Factors associated with alcohol use in pregnancy

	Drank alcohol in the last pregnancy (n = 700) n (%)		Intend to drink alcohol if planning pregnancy (n = 1103) n (%)		Intend to drink alcohol if pregnant (n = 1103) n (%)	
						
	Yes	No	Yes	No	Yes	No
**Participant characteristics**						
Age						
18 to 29 years	49 (38.9)	77 (61.1)	125 (28.5)	314 (71.5)	73 (16.6)	366 (83.4)
30 to 45 years	190 (33.1)	384 (66.9)	223 (33.6)	441 (66.4)	188 (28.3)	476 (71.7)
Birth history						
Nulliparous			109 (27.0)	294 (73.0)	56 (13.9)	347 (86.1)
Given birth	239 (34.1)	461 (65.9)	239 (34.1)	461 (65.9)	205 (29.3)	495 (70.7)
Highest education level						
Less than year 12	41 (26.3)	115 (73.7)	39 (22.4)	135 (77.6)	36 (20.7)	138 (79.3)
Completed year 12	42 (37.8)	69 (62.2)	72 (28.6)	180 (71.4)	50 (19.8)	202 (80.2)
Post school qualification below bachelor degree	83 (31.6)	180 (68.4)	123 (32.1)	260 (67.9)	87 (22.7)	296 (77.3)
Bachelor degree or higher	73 (42.9)	97 (57.1)	114 (38.8)	180 (61.2)	88 (29.9)	206 (70.1)
**Knowledge**						
"Drinking alcohol in pregnancy can affect the unborn child"						
Agree	206 (31.9)	439 (68.1)	305 (29.8)	718 (70.2)	223 (21.8)	800 (78.2)
Disagree	33 (60.0)	22 (40.0)	43 (53.8)	37 (46.3)	38 (47.5)	42 (52.5)
"Drinking alcohol during pregnancy can lead to life-long disabilities in a child"						
Agree	173 (30.7)	390 (69.3)	264 (28.6)	660 (71.4)	192 (20.8)	732 (79.2)
Disagree	66 (48.2)	71 (51.8)	84 (46.9)	95 (53.1)	69 (38.5)	110 (61.5)
**Attitudes**						
"Pregnant women should not drink alcohol"						
Agree	127 (23.5)	414 (76.5)	199 (22.5)	686 (77.5)	132 (14.9)	753 (85.1)
Disagree	112 (70.4)	47 (29.6)	149 (68.3)	69 (31.7)	129 (59.2)	89 (40.8)
Negative attitudes towards alcohol in pregnancy	120 (23.0)	401 (77.0)	185 (22.8)	627 (77.2)	128 (15.8)	684 (84.2)
Neutral or positive attitudes towards alcohol in pregnancy	116 (64.7)	63 (35.3)	130 (59.9)	87 (40.1)	110 (50.7)	107 (49.3)
**Alcohol consumption and smoking**						
Drank alcohol in last pregnancy						
Yes			176 (73.6)	63 (26.4)	187 (78.2)	52 (21.8)
No			29 (6.3)	432 (93.7)	52 (11.3)	409 (88.7)
Frequency of current alcohol consumption						
Less than 1 day per week	307 (78.9)	82 (21.1)	128 (22.5)	442 (77.5)	103 (18.1)	467 (81.9)
1 to 4 days per week	139 (53.5)	121 (46.5)	181 (39.2)	281 (60.8)	128 (27.7)	334 (72.3)
5 or more days per week	15 (29.4)	36 (70.6)	39 (54.9)	32 (45.1)	30 (42.3)	41 (57.7)
Maximum number of standard drinks in a day in the last 12 months						
0 drinks	4 (4.1)	94 (95.9)	19 (14.6)	111 (85.4)	20 (15.4)	110 (84.6)
1 to 2 drinks	46 (31.3)	101 (68.7)	53 (29.3)	128 (70.7)	43 (23.8)	138 (76.2)
3 to 6 drinks	109 (40.2)	162 (59.8)	123 (32.2)	259 (67.8)	96 (25.1)	286 (74.9)
7 or more drinks	80 (43.5)	104 (56.5)	153 (37.3)	257 (62.7)	102 (24.9)	308 (75.1)
Smoked in last pregnancy						
Yes	47 (41.6)	66 (58.4)	42 (37.2)	71 (62.8)	38 (33.6)	75 (66.4)
No	192 (32.7)	395 (67.3)	197 (33.6)	390 (66.4)	167 (28.4)	420 (71.6)
Intend to smoke if planning pregnancy						
Yes	20 (42.6)	27 (57.4)	27 (50.9)	26 (49.1)	23 (43.4)	30 (56.6)
No	219 (33.5)	434 (66.5)	321 (30.6)	729 (69.4)	238 (22.7)	812 (77.3)
Intend to smoke if pregnant						
Yes	23 (54.8)	19 (45.2)	27 (61.4)	17 (38.6)	26 (59.1)	18 (40.9)
No	216 (32.8)	442 (67.2)	321 (30.3)	738 (69.7)	235 (22.2)	824 (77.8)

The proportions for the factors associated with alcohol use in pregnancy are displayed in Table 3 and Table 4 presents the adjusted OR. The OR were adjusted for age, birth history and education level. Only the aOR are shown here but the crude OR (data not shown) were similar. Prior and current behaviour, and attitudes had strong associations with alcohol consumption in pregnancy. Drinking alcohol in the last pregnancy was a strong predictor of a woman's intention to drink alcohol if she were planning a pregnancy (aOR 29.6; 95% CI 19.1 to 45.7) or in a future pregnancy (aOR 43.9; 95% CI to 27.0 to 71.4). Women who disagreed with the statement that pregnant women should not drink were more likely to intend to drink alcohol during a future pregnancy (aOR 8.3; 95% CI 5.9 to 11.7), as were women who had neutral or positive attitudes towards alcohol consumption in pregnancy (aOR 5.1; 95% CI 3.6 to 7.1). Women with more frequent current alcohol use e.g. drinking on five or more days per week (aOR 3.2; 95% CI 1.9 to 5.4) and women who currently drink higher amounts of alcohol e.g. drinking seven or more drinks in a day (aOR 2.7; 95% CI 1.6 to 4.8) were more likely to intend to drink alcohol in a future pregnancy. Women who intended to smoke if pregnant were also more likely to intend to drink if pregnant. However, current smoking status was not significantly associated with intention to drink in pregnancy (data not shown). Other factors which were not associated with drinking in pregnancy were intention to become pregnant in the future, employment status and country of birth (data not shown) Marital status was significant on univariate analysis but was no longer significant once adjustment for the potential confounders was made (data not shown). Knowing that alcohol use in pregnancy can affect the unborn child and, that alcohol exposure in pregnancy can lead to lifelong disabilities in the child, were the only items of knowledge associated with alcohol use in pregnancy. Women who lacked this knowledge were more likely to intend to drink if pregnant. Women with higher levels of education, and women who had given birth previously were more likely to drink alcohol in pregnancy.

**Table 4:** Factors associated with alcohol use in pregnancy expressed as adjusted odds ratios and 95% confidence intervals

	Drank alcohol in the last pregnancy (n = 700)†	Intend to drink alcohol if planning a pregnancy (n = 1103)‡	Intend to drink alcohol if pregnant (n = 1103)‡
	**aOR (95% CI)**	**aOR (95% CI)**	**aOR (95% CI)**
**Participant characteristics**			
Age			
18 to 29 years	Reference	Reference	Reference
30 to 45 years	0.8 (0.6 to 1.3)	1.0 (0.7 to 1.4)	1.2 (0.9 to 1.8)
Birth history			
Nulliparous		Reference	Reference
Given birth		1.5 (1.1 to 2.2)*	2.5 (1.7 to 3.8)**
Highest education level			
Less than year 12	Reference	Reference	Reference
Completed year 12	1.8 (1.0 to 3.0)*	1.7 (116 to 2.7)*	1.5 (0.9 to 2.5)
Post school qualification below bachelor degree	1.2 ( 0.8 to 2.0)	1.8 (1.2 to 2.7)*	1.4 (0.9 to 2.1)
Bachelor degree or higher	2.2 (1.4 to 3.5)*	2.5 (1.6 to 3.9) **	2.2 (1.40 to 3.5)*
**Knowledge**			
Disagree that "Drinking alcohol in pregnancy can affect the unborn child"	3.8 (2.1 to 6.7)**	3.1 ( 2.0 to 5.0)**	3.6 (2.2 to 5.90)**
Disagree that "Drinking alcohol during pregnancy can lead to life-long disabilities in a child"	2.4 (1.6 to 3.5)**	2.4 (1.7 to 3.3)**	2.4 (1.7 to 3.4)**
**Attitudes**			
Disagree that "Pregnant women should not drink alcohol"	8.2 (5.5 to 12.2)**	7.5 (5.4 to 10.5)**	8.3 (5.9 to 11.7)**
Neutral or positive attitudes towards alcohol in pregnancy	6.2 (4.2 to 9.1)**	4.9 (3.6 to 6.8)**	5.1 (3.6 to 7.1)**
**Alcohol consumption and smoking**			
Drank alcohol in last pregnancy		29.6 (19.1 to 45.7)**	43.9 (27.0 to 71.4)**
Frequency of current alcohol consumption			
Less than 1 day per week	Reference	Reference	Reference
1 to 4 days per week	3.4 (2.4 to 4.8)**	2.3 (1.8 to 3.1)**	1.9 (1.4 to 2.6)**
5 or more days per week	9.3 (4.8 to 18.0)**	4.1 (2.5 to 6.9)**	3.2 (1.9 to 5.4)**
Maximum number of standard drinks in a day in the last 12 months			
0 drinks	Reference	Reference	Reference
1 to 2 drinks	11.1 (3.8 to 32.2)**	2.3 (1.3 to 4.1)*	1.6 (0.9 to 2.9)
3 to 6 drinks	17.8 (6.3 to 50.2)**	3.0 (1.7 to 5.1)**	2.0 (1.1 to 3.4)*
7 or more drinks	21.4 (7.5 to 61.4)**	4.5 (2.6 to 7.8)**	2.7 (1.6 to 4.8)**
Smoked in last pregnancy	1.8 (1.2 to 2.8)*	1.5 (1.0 to 2.3)	1.6 (1.0 to 2.5)*
Intend to smoke if planning pregnancy		2.5 (1.4 to 4.4)*	2.4 (1.4 to 4.4)*
Intend to smoke if pregnant		3.9 (2.0 to 7.3)**	4.7 (2.5 to 9.0)**

† Adjusted for age and highest education level

‡Adjusted for age, highest education level and birth history

*p < 0.05

**p < 0.001

On post hoc analysis of the association between level of education and current alcohol consumption, women who had a bachelor degree or higher were significantly more likely to drink at least one day per week (OR 1.8 95% CI 1.3 to 2.3). However, level of education remained a significant predictor of alcohol consumption in pregnancy after adjusting for frequency of current alcohol consumption.

## Discussion

In our survey, we identified the characteristics of women at high risk of drinking alcohol during pregnancy. Women's alcohol use in previous pregnancy, and their current alcohol and smoking behaviour were strong predictors of alcohol consumption in past pregnancy and intentions in a future pregnancy. Women's attitudes towards alcohol consumption in pregnancy were also strongly associated with alcohol consumption in pregnancy. Women's knowledge was a weaker predictor of alcohol consumption in pregnancy. Nevertheless, specific knowledge, including knowing that drinking alcohol in pregnancy can lead to lifelong disabilities in a child, was associated with alcohol consumption in pregnancy. We also identified groups that are at higher risk of alcohol consumption in pregnancy. These include women who have given birth previously, women who currently drink more frequently or have higher maximum alcohol consumption, and women with higher levels of education.

Nearly one quarter of Australian women intended to drink alcohol in a future pregnancy compared to fewer than ten per cent in the 2006 iteration of the Canadian survey [24]. This was the third Canadian survey and may reflect the effectiveness of Canadian FASD awareness and prevention campaigns [25]. The reported rate of alcohol consumption in pregnancy in our survey (34%) was almost half that reported in an earlier Australian survey [14]. Both surveys collected the alcohol consumption information retrospectively, but the previous survey collected data at twelve weeks postpartum, whereas in our survey the median time since birth of the last child was five years.

Our findings that university education [16,26,27], smoking [16,17,26-28], and current alcohol use [27] are associated with past and intended drinking in pregnancy confirm findings of previous studies. However, in a Canadian survey, women with a university education and younger women were more likely to report that they would not drink alcohol in a future pregnancy than women in our survey [28]. The differences regarding the influence of education level between Australia and Canada may again reflect greater awareness of FASD in Canada. In our survey, women with university education were more likely to drink in pregnancy but age was not a significant predictor of alcohol consumption in pregnancy. Age has been reported inconsistently as a predictor of drinking behaviour [23-26]. In our study, the association of older age with alcohol consumption in pregnancy disappeared with adjustment for having given birth previously.

Knowledge and attitudes are important potential influences of behaviour [19] but awareness of the effects of alcohol in pregnancy alone is not sufficient to change women's behaviour [18]. Attitudes were a much stronger predictor of alcohol consumption during pregnancy than knowledge. We have previously reported a disjunction between women's knowledge and attitudes towards alcohol consumption in pregnancy [23]. The disjunction is even more apparent when the strength of association between attitudes and alcohol consumption is compared to the relative weakness of the association between knowledge and alcohol consumption. The disjunction between knowledge and alcohol consumption may be related to the lack of evidence regarding harm to the fetus following exposure to low and moderate alcohol consumption in pregnancy [29]. It may be that women who have knowledge about alcohol in pregnancy and FASD, are more likely to be aware of the lack of evidence regarding the effects of low to moderate alcohol consumption in pregnancy and thus perceive that their risk is low if they consume alcohol at this level. Future studies would need to include more specific questions regarding the quantity of alcohol consumed in pregnancy and the women's knowledge regarding the effects of different levels of alcohol consumption in order to explore this further.

There appears to be a paradoxical relationship between women's level of education, their knowledge, and past and intended alcohol consumption in pregnancy. Women with higher levels of education have higher levels of knowledge [23] but despite this, are more likely to drink alcohol in pregnancy. Education level is an independent predictor of alcohol use in pregnancy but is not explained solely by women's current alcohol consumption. Excessive alcohol use by tertiary education students is well documented [30] and it may be that this engenders a more entrenched alcohol culture and more tolerant lifelong attitudes towards alcohol consumption in those who attend tertiary education institutions.

When discussing prevention of Fetal Alcohol Spectrum Disorders it is important to realise that women do not have sole responsibility for ensuring healthy offspring. Women's partners can have an important and positive influence. In a Danish study, paternal smoking was a strong predictor of maternal smoking during pregnancy [31]. However, in a US study, partners' alcohol use was not predictive of maternal alcohol consumption in pregnancy [32]. In our survey, whilst most women stated that it would make no difference to their behaviour, approximately one third of women stated that if their partner did not drink alcohol during pregnancy, they would be less likely to drink themselves.

The strengths of our study are that it is a national study with a large sample size and provides new information on the role of knowledge and attitudes regarding alcohol consumption in pregnancy and women's past and intended consumption of alcohol in pregnancy. A potential limitation of our study is that recall bias may have led to an underestimation of alcohol consumption in pregnancy. The study relied on women to report alcohol consumption retrospectively with an interval of up to 23 years between their last pregnancy and the time of the survey. Retrospective self-report of the quantity of alcohol consumed can be a reliable and valid method of measurement of alcohol consumption [33]. Although recall of alcohol consumption can deteriorate rapidly [34], retrospective self-reporting of alcohol consumption in pregnancy can lead to higher estimates than reporting during pregnancy [35,36]. We also used self-reporting of intention to drink in a future pregnancy which is a useful predictor of drinking in pregnancy [37], but is not the same as actually drinking during pregnancy. There was consistency of associations and strength of associations across the three time periods, i.e. past pregnancy, planning a future pregnancy and during a future pregnancy, which supports the validity of the survey findings.

As reported previously [23], there are limitations to the representativeness of this sample as can be seen by the fact that respondents were more likely to have been born in Australia, to have had a previous pregnancy, and to have achieved a higher level of education; and less likely to identify as Aboriginal or Torres Strait Islander than the general population. Unfortunately, we did not have any information about the women who chose not to complete the survey. The Electronic White Pages were chosen as the method of sampling as they provide an efficient method for surveying self-reported aspects of population health and have been shown to be accurate [38,39]. However, there has been a recent increase in the number of households without a listed number and individuals only using a mobile number which may or may not be listed in the Electronic White Pages. Populations which could be noticeably under-represented include younger people and people of lower socioeconomic status [38].

At the time this survey was conducted the National Health and Medical Research Council of Australia (NHMRC) recommended that women who are pregnant or might soon become pregnant may consider not drinking alcohol, should never become intoxicated and, if they choose to drink, should consume less than seven standard drinks in a week and no more than two standard drinks on one day [40]. These guidelines may have influenced women's responses to this survey. However, knowledge of these guidelines was poor among health professionals [41,42], and knowledge was also likely to be poor in the community. The NHMRC recommendations changed in 2009, and now state that not drinking alcohol is the safest option for women who are pregnant or planning pregnancy [43].

Interventions to prevent fetal alcohol exposure need to recognise the relative roles that knowledge and attitudes play in influencing women's alcohol consumption when planning a pregnancy or when pregnant. The perception that the risk to the fetus is low has previously been postulated as an underlying reason for multiparous women's increased likelihood of drinking alcohol during pregnancy [44]. The strong association between attitudes and alcohol consumption may be mediated by risk perception. Risk perception may also be affected by knowledge of severe and specific effects of alcohol consumption in pregnancy on the child e.g. lifelong disabilities. Our results did not show a relationship between non-specific knowledge and drinking behaviour, suggesting that interventions based on changing risk perception may be more successful in reducing fetal alcohol exposure than traditional awareness campaigns.

## Conclusion

In summary, we found drinking behaviour and attitudes were the strongest predictors of women's intended alcohol consumption when planning a pregnancy or pregnant. We identified groups at increased risk of drinking alcohol during pregnancy, including women with university education, multiparous women, and women with current high risk drinking, indicating a need for targeted interventions to change their perception of risk and hence their behaviour. Both community and individual interventions should take into consideration the role partners can play and the frequent co-occurrence of smoking and alcohol consumption. This reinforces the need for an integrated approach to promoting a healthy pregnancy including timely antenatal care, appropriate maternal nutrition, folate supplementation and efforts to reduce the use of alcohol, tobacco and illicit drug use in pregnancy. The opportunity to influence women who are planning pregnancy should be seized as this may be the best chance public health systems have to prevent fetal alcohol exposure. The questions used in this survey, specifically drinking in last pregnancy and attitudes towards alcohol consumption in pregnancy, could be examined in futures studies for their utility in the clinical setting to identify women at risk of drinking alcohol during pregnancy.

## Conflict of interest statement

The authors declare that they have no competing interests.

## Authors' contributions

EP, JP, NH, HD, AB, CO'L, CB and EJE conceived and designed the study; EP conducted the analysis and wrote the first draft of the paper. All authors contributed to writing the paper and approved the final version for publication. EP is guarantor for the study.

## Pre-publication history

The pre-publication history for this paper can be accessed here:

http://www.biomedcentral.com/1471-2458/11/584/prepub

## Supplementary Material

Additional file 1 **Alcohol and Pregnancy Questionnaire**. This file contains the questionnaire used for the survey. Click here for file
