# Supplementary material for: Attitudes and behaviour predict women's intention to drink alcohol during pregnancy: the challenge for health professionals
Source: BMC Public Health. 2011 Jul 22;11:584. doi: 10.1186/1471-2458-11-584 (PMC3155919; doi:10.1186/1471-2458-11-584)
Supplement: Additional file 1 — Alcohol and Pregnancy Questionnaire. This file contains the questionnaire used for the survey. [file 1471-2458-11-584-S1.DOC]

# Telethon Institute for Child Health Research

**Alcohol and Pregnancy**

**Questionnaire**

**Introduction**

Good morning/afternoon/evening, my name is _______________________ and I am calling from The Survey Research Centre at The University of WA. We are conducting a survey on behalf of the Institute for Child Health Research about some important health issues. Would it be possible for me to speak with a woman aged between 18 and 45 in your household?

1 - Yes

2 - No

**IF NO, POLITELY TERMINATE**

**IF YES, ASK TO SPEAK TO THAT PERSON, AND CONTINUE.**

**WHEN SPEAKING TO THE CORRECT AGED FEMALE – IF THERE IS ONE IN THAT HOUSEHOLD.**

We are conducting a survey about some important health issues and we are very interested in your opinion. I would like to ask you to take part in a survey. I can assure you that all information given will remain confidential and no individual answers will be passed on. Instead, all responses to this survey will be collected together and will be used to create informational materials that will be used to help all families.

Is now a convenient time to do the survey?

If not, then we are very happy to make an appointment to call back.

Before we begin, can I just ask:

B) Are you currently pregnant?

1 - Yes

2 - No

**IF YES, POLITELY TERMINATE**

**IF NO, CONTINUE.**

**Behaviours to increase the likelihood of having a healthy baby**

**Top of the mind awareness behaviours**

1. Thinking about healthy babies and children, what do you think are the most important things that pregnant women can do to increase the likelihood that their unborn child will be healthy? What things come to mind as important? **DO NOT READ. CODE ALL THAT APPLY. FIRST MENTION. SECOND MENTION, THIRD MENTION. ARE THERE ANY OTHERS?**
   - - - 1. Visit doctor/health professional/Aboriginal Medical Service
         2. Eat well/good nutrition
         3. Increase exercise/physical activity
         4. Reduce exercise/physical activity
         5. Cut down/stop smoking
         6. Cut down/stop alcohol use
         7. Cut down/stop drug use (marijuana, crack, heroin, etc.)
         8. Avoid stress
         9. Avoid environmental pollution
         10. Avoid second hand smoke
         11. Positive mental attitude
         12. Learn about infant care
         13. Take pre-natal class
         14. Talk to friends/family/social support
         15. Take folic acid/folate
         16. Get rest/sleep
         17. Other (SPECIFY)
         18. DK/NA
2. I’m going to read some things that pregnant women might do to increase the likelihood that their unborn child will be healthy. In your opinion, is each of the following very important, important, not very important or not at all important to do? **READ.**
3. Visit a doctor or health professional
   - 1. Very important
     2. Important
     3. Not very important
     4. not at all important
     5. DK/NA
4. Take folate or folic acid before and during the pregnancy
5. Cut down or stop smoking
6. Cut down or stop drinking alcohol

**Awareness of the effects of alcohol during pregnancy and fetal alcohol spectrum disorders**

1. Have you ever heard of any effects on pregnancy or the unborn child which are caused by drinking alcohol during pregnancy? **DO NOT READ**

1 - Yes **ASK Q3b**

2 - No **GO TO Q4a**

3 - DK/NA **GO TO Q4a**

1. Can you tell me what are the effects on the pregnancy or unborn child of drinking alcohol during pregnancy? **PROBE FOR COMPLETE RESPONSE.** What can happen to the unborn child following drinking alcohol during pregnancy? **DO NOT READ**
2. Fetal Alcohol Syndrome **SKIP Q4a**
3. Fetal Alcohol Spectrum Disorders **SKIP Q4c**
4. Fetal Alcohol Effects **SKIP Q4e**
5. Alcohol Related Birth Defects **SKIP Q4g**
6. Alcohol Related Neurodevelopmental Disorder **SKIP Q4i**
7. Baby addicted/experiences withdrawal
8. Harmful/ill effects
9. Delayed development
10. Physical disorders
11. Learning disabilities
12. Causes birth defects/deformities
13. Brain damage
14. Mental disorders
15. Cranial/facial deformities
16. Lower IQ/retardation
17. Behavioural problems
18. Low birth weight
19. Attention Deficit Disorder
20. Born with alcohol in its system
21. Growth problems
22. Premature birth
23. Miscarriage
24. Stillbirth
25. Other
26. DK/NA

**IF NONE OF THE ANSWERS CODED 1-5 ARE MENTIONED, GO TO Q4a**

1. Have you ever heard of Fetal Alcohol Syndrome? **DO NOT READ.**
2. Yes **ASK Q4b**
3. No **GO TO Q4c**
4. DK/NA **GO TO Q4c**
5. Can you tell me what Fetal Alcohol Syndrome is? **PROBE FOR COMPLETE RESPONSE.**

What happens with Fetal Alcohol Syndrome? **DO NOT READ.**

- - 1. Effects of alcohol on fetus
    2. Baby addicted/experiences withdrawal
    3. Harmful/ill effects
    4. Delayed development
    5. Physical disorders
    6. Learning disabilities
    7. Causes birth defects/deformities
    8. Brain damage
    9. Mental disorders
    10. Cranial/facial deformities
    11. Lower IQ/retardation
    12. Behavioural problems
    13. Low birth weight
    14. Attention Deficit Disorder
    15. Born with alcohol in its system
    16. Growth problems
    17. Premature birth
    18. Miscarriage
    19. Stillbirth
    20. Other
    21. DK/NA

1. Have you ever heard of Fetal Alcohol Spectrum Disorders? **DO NOT READ.**

1 - Yes **ASK Q4d**

2 - No **GO TO Q4e**

3 - DK/NA **GO TO Q4e**

1. Can you tell me what Fetal Alcohol Spectrum Disorders are? **PROBE FOR COMPLETE RESPONSE.**

What happens with Fetal Alcohol Spectrum Disorders? **DO NOT READ.**

- - 1. Effects of alcohol on fetus
    2. Baby addicted/experiences withdrawal
    3. Harmful/ill effects
    4. Delayed development
    5. Physical disorders
    6. Learning disabilities
    7. Causes birth defects/deformities
    8. Brain damage
    9. Mental disorders
    10. Cranial/facial deformities
    11. Lower IQ/retardation
    12. Behavioural problems
    13. Low birth weight
    14. Attention Deficit Disorder
    15. Born with alcohol in its system
    16. Growth problems
    17. Premature birth
    18. Miscarriage
    19. Stillbirth
    20. Other
    21. DK/NA

1. Have you ever heard of Fetal Alcohol Effects? **DO NOT READ.**

1 - Yes **ASK Q4f**

2 - No **GO TO Q4g**

3 - DK/NA **GO TO Q4g**

1. Can you tell me what Fetal Alcohol Effects is? **PROBE FOR COMPLETE RESPONSE.**

What happens with Fetal Alcohol Effects? **DO NOT READ.**

- - 1. Effects of alcohol on fetus
    2. Baby addicted/experiences withdrawal
    3. Harmful/ill effects
    4. Delayed development
    5. Physical disorders
    6. Learning disabilities
    7. Causes birth defects/deformities
    8. Brain damage
    9. Mental disorders
    10. Cranial/facial deformities
    11. Lower IQ/retardation
    12. Behavioural problems
    13. Low birth weight
    14. Attention Deficit Disorder
    15. Born with alcohol in its system
    16. Growth problems
    17. Premature birth
    18. Miscarriage
    19. Stillbirth
    20. Other
    21. DK/NA

1. Have you ever heard of Alcohol Related Birth Defects? **DO NOT READ.**

1 - Yes **ASK Q4h**

2 - No **GO TO Q4i**

3 - DK/NA **GO TO Q4i**

1. Can you tell me what Alcohol Related Birth Defects are ? **PROBE FOR COMPLETE RESPONSE.**

What happens with Alcohol Related Birth Defects? **DO NOT READ.**

- - 1. Effects of alcohol on fetus
    2. Baby addicted/experiences withdrawal
    3. Harmful/ill effects
    4. Delayed development
    5. Physical disorders
    6. Learning disabilities
    7. Causes birth defects/deformities
    8. Brain damage
    9. Mental disorders
    10. Cranial/facial deformities
    11. Lower IQ/retardation
    12. Behavioural problems
    13. Low birth weight
    14. Attention Deficit Disorder
    15. Born with alcohol in its system
    16. Growth problems
    17. Premature birth
    18. Miscarriage
    19. Stillbirth
    20. Other
    21. DK/NA

1. Have you ever heard of Alcohol Related Neurodevelopmental Disorder? **DO NOT READ.**

1 - Yes **ASK Q4j**

2 - No **GO TO Q5**

3 - DK/NA **GO TO Q5**

1. Can you tell me what Alcohol Related Neurodevelopmental Disorder is ? **PROBE FOR COMPLETE RESPONSE.** What happens with Alcohol Related Neurodevelopmental Disorder? **DO NOT READ.**
   - 1. Effects of alcohol on fetus
     2. Baby addicted/experiences withdrawal
     3. Harmful/ill effects
     4. Delayed development
     5. Physical disorders
     6. Learning disabilities
     7. Causes birth defects/deformities
     8. Brain damage
     9. Mental disorders
     10. Cranial/facial deformities
     11. Lower IQ/retardation
     12. Behavioural problems
     13. Low birth weight
     14. Attention Deficit Disorder
     15. Born with alcohol in its system
     16. Growth problems
     17. Premature birth
     18. Miscarriage
     19. Stillbirth
     20. Other
     21. DK/NA

**Knowledge and Attitudes about Drinking Alcohol in Pregnancy**

I’m going to ask you a few questions about drinking alcohol during pregnancy.

1. Have you ever heard of a standard drink of alcohol?
   - - - 1. Yes **GO TO Q6**
         2. No **] READ: A STANDARD DRINK IS …**
         3. DK/NA **]**
2. A Please tell me if you strongly agree, agree, disagree or strongly disagree with the following statements…

**READ**

1. Pregnant women should not drink alcohol.

1 – Strongly agree

2 - Agree

3 - Disagree

4 – Strongly disagree

5 - DK/NA

1. Pregnant women should drink less than seven standard alcoholic drinks each week. A standard drink is …
2. It is ok for pregnant women to drink three or four standard alcoholic drinks on one day.
3. It is ok for pregnant women to become intoxicated.
4. Drinking alcohol during pregnancy can affect the unborn child.
5. The more alcohol a pregnant woman drinks, the more likely that the unborn child will be harmed.
6. Drinking alcohol during pregnancy can lead to life-long disabilities in a child.
7. Women are aware of the effects that drinking alcohol during pregnancy can have on the unborn child
8. Members of the community are concerned about women drinking alcohol during pregnancy.
9. Information should be readily available to women about the effect that drinking alcohol during pregnancy may have on the unborn child.
10. Health professionals should ask pregnant women about how much and how often they drink alcohol.
11. Health professionals should advise women who are pregnant or who are thinking of becoming pregnant to give up drinking alcohol.

6) B a) If you saw a pregnant woman drinking alcohol, how would you feel? **DO NOT READ**

1 - Angry

2 – Annoyed

3 – Upset

4 - Disgusted

5 – Other (specify) ___________________________________

b) On a scale of one to five, one being low and five being high, how <state 6Ba> would you feel?

_______________

**Recall of information about the effects of alcohol use**

1. Have you seen or heard about any information on the effects of drinking alcohol during pregnancy on the unborn child? **DO NOT READ**

1 - Yes **ASK Q7b**

2 - No **GO TO Q8a**

3 - DK/NA **GO TO Q8a**

1. What kind of information was it? **DO NOT READ. CODE ALL THAT APPLY**
2. Brochure/pamphlet
3. Media programs/articles in newspapers/magazines
4. Books
5. Television advertising
6. Magazine advertising
7. Infant care groups/classes
8. Poster
9. Doctor/health care professional
10. Radio
11. School/special classes
12. Personal experience/word of mouth
13. Alcohol bottles/alcohol vendors
14. Work
15. Presentation/seminar
16. Internet
17. Other **(specify)**
18. DK/NA

**Support for initiatives to provide information about the effects of drinking alcohol during pregnancy.**

1. What for you would be the best source of information about the effects of drinking alcohol during pregnancy on the unborn child? **DO NOT READ**.
2. Doctor/doctor's office/Aboriginal medical service
3. Health clinic/hospital
4. Prenatal/antenatal class
5. Pharmacy/chemist
6. Workplace
7. Through friends/family
8. Books/magazines
9. Internet sources
10. TV programs
11. Flyers and pamphlets
12. Public health organizations/programs
13. Library
14. Newspapers
15. TV advertisements
16. Other media
17. School
18. Alcohol bottles/alcohol vendors
19. Posters/billboards
20. Journals
21. Others **(SPECIFY)**
22. DK/NA
23. Are there any other good sources for you to find this information? **DO NOT READ. CODE ALL THAT APPLY.**
24. Doctor/doctor's office/Aboriginal medical service
25. Health clinic/hospital
26. Prenatal/antenatal class
27. Pharmacy/chemist
28. Workplace
29. Through friends/family
30. Books/magazines
31. Internet sources
32. TV programs
33. Flyers and pamphlets
34. Public health organizations/programs
35. Library
36. Newspapers
37. TV advertisements
38. Other media
39. School
40. Alcohol bottles/alcohol vendors
41. Posters/billboards
42. Journals
43. Others **(SPECIFY)**
44. DK/NA
45. Do you strongly approve, approve, disapprove or strongly disapprove of each of the following: **READ**
46. Labels on alcohol products warning about the effects of drinking alcohol during pregnancy.
47. Strongly approve
48. Approve
49. Disapprove
50. Strongly disapprove
51. DK/NA
52. Signs in restaurants, bars and clubs warning about the effects of drinking alcohol during pregnancy.
53. Government-sponsored advertising warning about the effects of drinking alcohol during pregnancy.
54. Messages on alcohol advertising warning about the effects of drinking alcohol during pregnancy.
55. Now, I would like you to consider some ways of informing women about the effects of drinking alcohol during pregnancy on the unborn child.

Would the following ways be a very effective, effective, not very effective, or not at all effective way to reach women on this subject? **READ**

1. A website with information about the subject.
2. Very effective
3. Effective
4. Not very effective
5. Not at all effective
6. DK/NA
7. Sending information materials to doctors and health professionals so they can inform their patients
8. Posters and brochures in waiting rooms and clinics
9. Inserts and advertisements in magazines and newspapers
10. Advertisements on buses and bus shelters
11. Posters and brochures in pharmacies and chemists
12. Television advertisements
13. Radio advertisements
14. School education programmes

**Tobacco and Alcohol consumption**

Now I’d like to ask some questions about your lifestyle.

1. Do you consider yourself…? **READ**
   - - - 1. A non-smoker **] If EITHER OF THESE OPTIONS SELECTED, Q21b AND Q21d**
         2. An ex-smoker **]**
         3. A smoker
         4. DK/NA
2. Now thinking about alcohol, have you ever tried alcohol? **DO NOT READ**
   - - - 1. Yes **GO TO Q12b**
         2. No **GO TO Q16**
         3. DK/NA **GO TO Q16**
3. Have you had an alcoholic drink of any kind in the last 12 months? **DO NOT READ**
4. Yes **GO TO Q11c**
5. No **GO TO Q15**
6. DK/NA **GO TO Q15**
7. In the last 12 months, how often did you have an alcoholic drink of any kind? **READ.**
8. Every day
9. 5 to 6 days a week
10. 3 to 4 days a week
11. 1 to 2 days a week
12. 2 to 3 days a month
13. about 1 day a month
14. Less often
15. No longer drink
16. On a day that you have an alcoholic drink, how many standard drinks do you usually have? **READ.** A standard drink is …
17. 13 or more drinks **DELETE Q13b-f**
18. 11 to 12 drinks **DELETE Q13b-f**
19. 7 to 10 drinks **DELETE Q13c-f**
20. 5 to 6 drinks **DELETE Q13d-f**
21. 3 to 4 drinks **DELETE Q13e-f**
22. 1 to 2 drinks **DELETE Q13f**
23. less than 1 drink
24. In the last 12 months, have you ever had the following number of alcoholic standard drinks in a day? **READ.**
25. 20 or more standard drinks a day
    1. Yes
    2. No
26. 11 to 19 standard drinks a day
27. 7 to 10 standard drinks a day
28. 5 to 6 standard drinks a day
29. 3 to 4 standard drinks a day
30. 1 to 2 standard drinks a day
31. How many alcoholic standard drinks did you have yesterday? _____
32. At the present time, do you consider yourself:
33. a non-drinker **] If EITHER OF THESE OPTIONS SELECTED, SKIP Q21a AND Q21c**
34. an ex-drinker **]**
35. an occasional drinker
36. a light drinker
37. a social drinker
38. a heavy drinker
39. a binge drinker
40. As far as you know, is the number of standard drinks shown on cans and bottles of alcoholic beverages?
    - - - 1. Yes
          2. No
          3. DK/NA
41. Have you ever heard of the Australian Alcohol Guidelines?
    - - - 1. Yes
          2. No
          3. DK/NA
42. How many standard drinks do you believe a woman could drink every day for many years without adversely affecting her health? ________

**Women and their partners**

**Women – alcohol use during pregnancy**

1. Have you ever given birth to a child? **DO NOT READ**
2. Yes **ASK Q19b**
3. No **GO TO Q20a**
4. DK/NA **GO TO Q20a**
5. How many children have you given birth to? **DO NOT READ**
6. 1
7. 2
8. 3
9. 4
10. 5
11. 6 or more
12. DK/NA
13. In what year was your last child born? _________

d) Thinking back to when you were pregnant with your last child that was born in <insert>, did you

1 not drink alcohol

2 - not change your alcohol drinking

3 - cut back on your alcohol drinking

4 - stop drinking alcohol

e) Again, thinking back to when you were pregnant with your last child that was born in <insert>, did you

1 not smoke

2 - not change your smoking

3 - cut back on your smoking

4 - stop smoking

1. Thinking about yourself, do you think you might become pregnant within the next two years? **DO NOT READ**
2. Yes, might become pregnant within the next two years **GO TO Q21a**
3. No **GO TO Q20b**
4. DK/NA **GO TO Q20b**
5. Do you think you might become pregnant at some point in the future? **DO NOT READ**
6. Yes **GO TO Q21a**
7. No **GO TO Q21a**
8. DK/NA **GO TO Q21a**
9. If you were planning to become pregnant, would you… **READ**
10. not change your alcohol drinking
11. cut back on your alcohol drinking
12. stop drinking alcohol
13. If you were planning to become pregnant would you … **READ**
14. not change your smoking
15. cut back on your smoking
16. stop smoking
17. If you were pregnant, would you… **READ**
18. not change your alcohol drinking
19. cut back on your alcohol drinking
20. stop drinking alcohol
21. If you were pregnant, would you… **READ**
22. not change your smoking
23. cut back on your smoking
24. stop smoking

**Influence of partner on women’s alcohol use during pregnancy**

1. Thinking about yourself, if you were pregnant, would you be more likely to drink alcohol, less likely to drink alcohol or would it make no difference to your alcohol drinking in each of the following situations. **READ**
2. If your spouse or partner continued to drink alcohol during your pregnancy?
3. More likely
4. Less likely
5. No difference
6. If your spouse or partner encouraged you to stop or cut back your alcohol drinking during your pregnancy?
7. If your spouse or partner were to offer you alcohol during your pregnancy?
8. If your spouse or partner stopped drinking alcohol during your pregnancy?

**Demographic information**

Finally, a few questions for statistical purposes. Please be assured that all your responses will be kept entirely anonymous and absolutely confidential.

1. What was your age at your last birthday____
2. Which one of the following best describes your present marital status? **READ**
   - - - 1. Never married
         2. Widowed
         3. Divorced
         4. Separated but not divorced
         5. Married (including de facto, or living with life partner)
3. Are you of Aboriginal or Torres Strait Islander origin? **DO NOT READ**
   - - - 1. No
         2. Yes, Aboriginal
         3. Yes, Torres Strait Islander
         4. Yes, both Aboriginal and Torres Strait Islander
4. In which country were you born? **DO NOT READ**
   - - - 1. Australia **GO TO Q28**
         2. China
         3. Germany
         4. Greece
         5. Hong Kong
         6. India
         7. Ireland (Republic of)
         8. Italy
         9. Lebanon
         10. Malaysia
         11. Malta
         12. Netherlands
         13. New Zealand
         14. Philippines
         15. Poland
         16. South Africa
         17. Turkey
         18. United Kingdom (England, Wales, Northern Ireland)
         19. USA
         20. Vietnam
         21. Yugoslavia (the former)
         22. Other (specify)
5. In what year did you first arrive in Australia to live here for one year or more?

___________

Not applicable – will be in Australia for less than one year.

1. What is the postcode of your usual residence?__________
2. Which of the following best describes your current employment status? **READ.**
   - - - 1. Self-employed **GO TO Q31**
         2. Employed for wages, salary or payment in kind **GO TO Q31**
         3. Unemployed and looking for work **GO TO Q30**
         4. Engaged in home duties **GO TO Q30**
         5. A student **GO TO Q30**
         6. Retired or on a pension **GO TO Q30**
         7. Unable to work **GO TO Q30**
         8. Other **GO TO Q30**
3. Have you ever been in paid work?
   - - - 1. Yes
         2. No
4. What is the highest year of primary or secondary school you have completed? **DO NOT READ.**
   - - - 1. Did not go to school
         2. Year 6 or below
         3. Year 7 or equivalent
         4. Year 8 or equivalent
         5. Year 9 or equivalent
         6. Year 10 or equivalent
         7. Year 11 or equivalent
         8. Year 12 or equivalent
5. Have you completed a trade certificate or other education qualification? **DO NOT READ.**
   - - - 1. Yes
         2. No
6. What is the highest qualification that you have obtained? **DO NOT READ.**
   - - - 1. Trade certificate
         2. Non-trade certificate
         3. Associate Diploma
         4. Undergraduate Diploma
         5. Bachelor Degree
         6. Master’s Degree, Postgraduate Degree or Postgraduate Diploma
         7. Doctorate

**POLITELY TERMINATE.**

This is the end of the interview. Thank you very much for your time. If you would like more information on this topic or to receive some information about alcohol and pregnancy please call <insert name> on <insert number> or <insert website>. Just to remind you that my name is ________________ from the Survey Research Centre at The University of Western Australia. If you have any other questions about this research you can telephone our office on 9347 4054 and ask for the supervisor on duty.
